# A New Remedy, and a New Indication for an Old One

**Published:** 1889-06

**Authors:** R. B. Johnstone


### A NEW REMEDY, AND A NEW INDICATION FOR AN OLD ONE.

VITRUM (crown glass).—Bone diseases, when the discharge is thin, watery, and stinking, *much fine grinding, grating* pain, like rubbing from sand-paper or grit (cured in a case of Pott's disease after Silicea failed to make any impression).

THUJA (high).—In women who have a tendency to hernia on *left side* after labor, especially when the feet get sore and swell (sycotic history).

THUJA.—When babies cry much the umbilicus protrudes, grows red and sore, especially when the father has a sycotic history.

Infantile hernia on *left* side—inguinal—child cries all the time, and is only quiet when the left inguinal region is relieved from pressure or when the thigh is flexed upon the abdomen.

R. B. JOHNSTONE.

